# Support Needs of Family Caregivers During Tracheostomy Transitions: A Mixed‐Methods Study

**DOI:** 10.1002/nop2.70731

**Published:** 2026-08-07

**Authors:** Ying Wei, Ying Sun, Zhen Wang, Muli Zhu

**Affiliations:** ^1^ Jinling Hospital, Affiliated Hospital of Medical School, Nanjing University Nanjing Jiangsu China

**Keywords:** care transitions, caregivers, critical care nursing, discharge planning, mixed‐methods research, tracheostomy, tracheostomy care education

## Abstract

**Aim:**

To explore the support needs of family caregivers during the transition of tracheostomized patients from the respiratory intensive care unit (RICU) to the general ward or home and to provide evidence for nurse‐led, family‐centred transitional care.

**Design:**

This was a sequential explanatory mixed‐methods study, beginning with a quantitative survey and followed by qualitative interviews.

**Methods:**

In the quantitative phase, 128 family caregivers of tracheostomized patients completed the Family Needs in ICU Transition Scale within 24–48 h before transfer from the RICU to the general ward or home. Descriptive statistics were used to summarize overall needs, domain scores and item rankings. In the qualitative phase, semi‐structured interviews were undertaken with 11 caregivers purposively selected from the highest‐scoring 30% of the quantitative sample based on overall need scores. Interview data were analysed using Colaizzi's seven‐step phenomenological method. The qualitative findings helped to further interpret the quantitative results.

**Results:**

Family caregivers reported a high level of support needs overall, with a mean item score of 4.10 ± 0.85. The highest scoring domains were caregiving skills (4.25 ± 0.80), information needs (4.18 ± 0.84) and communication with healthcare providers (4.15 ± 0.81). Among this high‐need subgroup, five themes emerged from the interviews: the need for timely and detailed information before and during transfer, the need for professional guidance on post‐transfer care skills, the expectation of ongoing communication and information transparency, the request for caregiver educational materials and the desire for emotional support and encouragement.

**No Patient or Public Contribution:**

Patients and the public were not involved in the design, conduct, reporting or dissemination of this research.

## 1 Introduction

Tracheostomy is a common and critical intervention for maintaining airway patency in critically ill patients admitted to respiratory intensive care units (RICUs). It facilitates airway management, supports prolonged mechanical ventilation and often accelerates the weaning process (Durbin Jr. 2010; Stelfox et al. 2009). Although advances in critical care have improved survival rates among tracheostomized patients, their post‐RICU recovery remains complex, often involving significant family caregiver participation and support. The transition from a highly monitored RICU environment to general wards or home care constitutes a particularly vulnerable period for both patients and caregivers.

For tracheostomized patients, the transition from the RICU to the general ward or home represents a critical stage during which family caregivers are expected to assume increasing responsibilities in daily care, symptom observation, communication with healthcare staff and basic tracheostomy‐related care. Caregiving responsibilities may differ by transition destination: after transfer to the general ward, caregivers are mainly involved in bedside observation, basic tracheostomy care and communication with the ward healthcare team; after transfer directly home, they may need to undertake broader responsibilities, including tracheostomy care, nutritional support, observation of warning signs and communication with healthcare providers after discharge (Danesh et al. 2022). During this time, family caregivers, who often assume the primary role in providing daily care, face considerable physical, emotional and psychological challenges (Griffith et al. 2022; Gunnlaugsdóttir et al. 2024). Particularly for tracheostomized patients, the complexity of airway management, nutritional support and mobility assistance imposes substantial demands on caregivers. Without adequate preparation and nursing support, caregivers may experience heightened stress, anxiety and a sense of helplessness, which can adversely affect patient recovery (Picard et al. 2025).

Family caregivers are indispensable to the continuity of post‐ICU care. Nevertheless, numerous studies have highlighted significant gaps in caregiver education, emotional support and skill preparedness during ICU discharge planning (Picard et al. 2025; Al‐Mutair et al. 2013). Caregivers frequently report feelings of unpreparedness, insufficient communication with healthcare providers and lack of clear guidance regarding the patient's evolving care needs (Stricker et al. 2009). These challenges are even more pronounced among caregivers of tracheostomized patients, given the technical nature of airway management and the potential for life‐threatening complications if care is improperly administered.

Despite widespread recognition of family engagement as a core element of patient‐centered care, the specific needs of caregivers managing tracheostomy during the RICU transition remain poorly understood. Existing research has predominantly focused on general ICU populations or post‐ICU syndrome without distinguishing the unique burdens faced by caregivers managing tracheostomy care (Hiser et al. 2023). As a result, there is a critical knowledge gap regarding how best to support these caregivers during the transition phase to optimise both patient outcomes and caregiver well‐being.

Understanding the comprehensive support needs of family caregivers during this transition is vital to inform the design of effective nurse‐led transitional care. Tailored transitional care programs have the potential to reduce hospital readmissions, strengthen caregiver competence and facilitate smoother reintegration of patients into community or home settings (Choi et al. 2016). Furthermore, identifying specific unmet needs in areas such as technical care skills, communication with healthcare providers, emotional resilience and access to information can help guide the development of structured educational and psychosocial support resources.

To address these gaps, this study adopted a sequential explanatory mixed methods design to investigate the support needs of family caregivers during the transition of tracheostomized patients from the RICU. The quantitative phase aimed to systematically assess the levels and domains of caregiver needs using a validated scale, while the qualitative phase sought to further explore caregivers' experiences, challenges and expectations in relation to the quantitative findings. This study aimed to provide empirical evidence to inform structured, family‐centred, nurse‐led transitional care strategies for tracheostomized patients.

## 2 Methods

### 2.1 Study Design

**FIGURE 1:**
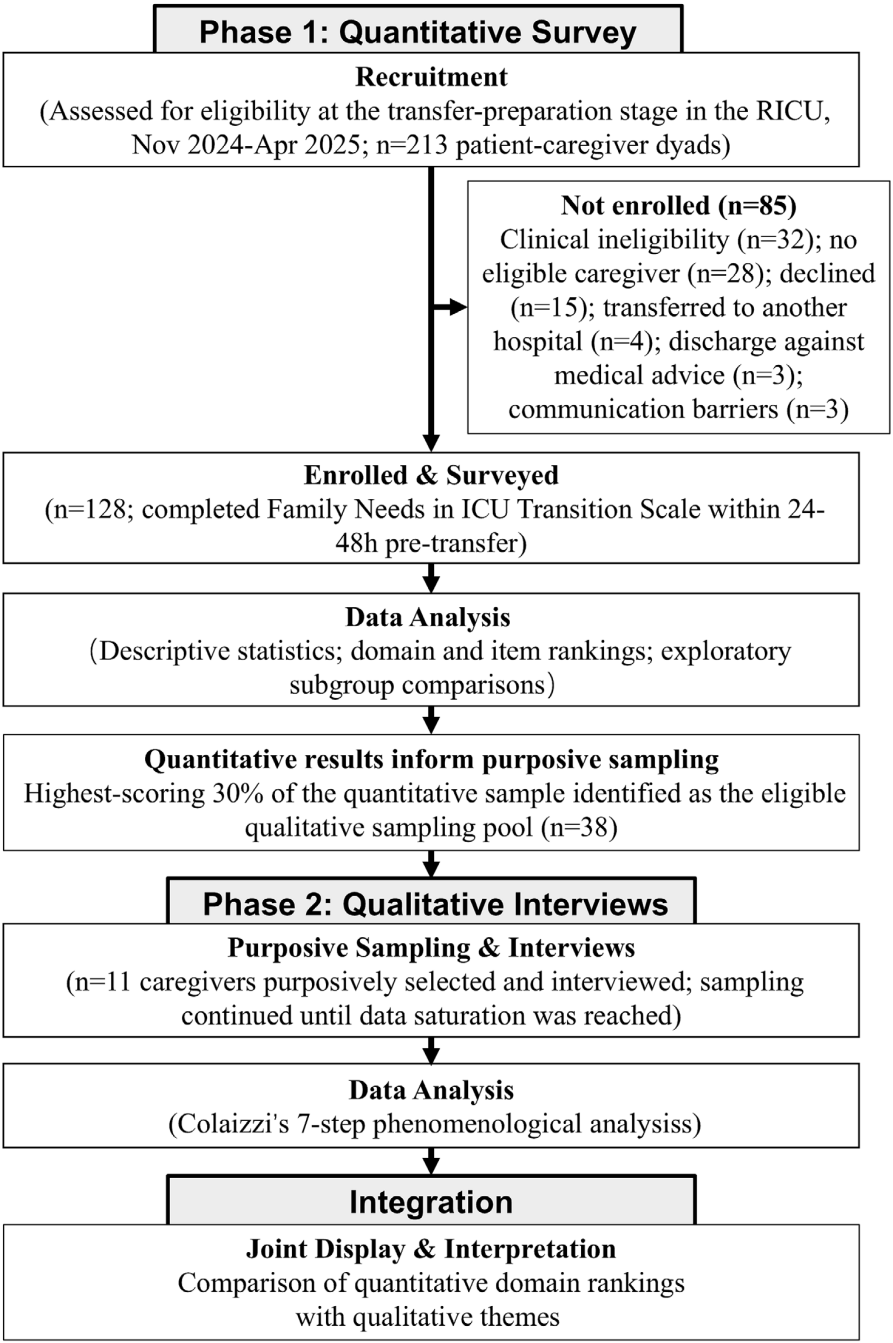
Sequential explanatory mixed‐methods study flow diagram. The figure illustrates quantitative recruitment and analysis, identification of the high‐need qualitative sampling pool, qualitative interviews and analysis and integration through joint display and interpretation. All reasons for non‐enrollment and their corresponding numbers are presented in the figure. RICU, respiratory intensive care unit.

This study employed a sequential explanatory mixed‐methods design, integrating quantitative and qualitative approaches across two distinct phases. The quantitative phase was conducted first to systematically assess the levels and characteristics of family caregivers' support needs. Subsequently, the qualitative phase explored participants' experiences and perspectives in more detail to complement and explain the quantitative findings. This design was selected to provide a comprehensive understanding of caregivers' needs during the transition of tracheostomized patients from the RICU. Integration occurred at the methods, sampling and interpretation levels: the qualitative phase was informed by the quantitative results, participants for interviews were purposively selected from caregivers reporting the highest levels of overall perceived need and the final interpretation compared areas of convergence between the two datasets. The study followed the STROBE guidelines for the quantitative observational component and the COREQ checklist for reporting qualitative interviews (von Elm et al. 2007; Tong et al. 2007). The overall study process is presented in Figure 1.

### 2.2 Setting and Participants

The study was conducted in the Respiratory Intensive Care Unit (RICU) of a tertiary general hospital in China. The RICU is a 20‐bed unit that provides specialized care for critically ill patients with respiratory diseases, including patients requiring tracheostomy. It is staffed by five attending physicians and 25 specialized nurses and provides 24‐h medical and nursing care. The usual discharge or transfer process for tracheostomized patients includes assessment of eligibility for transfer, preliminary caregiver training in basic tracheostomy care, handover of relevant medical and care information, development of a post‐transfer care plan and final discharge guidance. Family caregivers are allowed to participate in basic daily care, such as feeding and repositioning, during the patient's stay in the RICU. For tracheostomized patients, caregivers receive bedside guidance from specialized nurses on basic tracheostomy‐related care, such as airway humidification and dressing change, after successful ventilator weaning. Caregiver responsibilities differed by transition destination: caregivers of patients transferred to the general ward were mainly responsible for bedside observation, basic tracheostomy care and communication with the ward healthcare team. By contrast, caregivers of patients discharged directly home were expected to undertake broader responsibilities, including tracheostomy care, nutritional support, observation of warning signs and coordination with community healthcare providers after discharge. The study was conducted between November 2024 and April 2025.

#### 2.2.1 Quantitative Phase

Family caregivers of tracheostomized patients preparing for RICU discharge were recruited using consecutive sampling. Eligible caregivers were enrolled consecutively during the study period at the transfer‐preparation stage. Eligibility criteria included: (1) being the primary caregiver of a tracheostomized patient who had been successfully weaned from invasive mechanical ventilation for at least 72 consecutive hours, defined as remaining off invasive mechanical ventilation during that period and being considered clinically stable for transfer by the treating team; (2) actively participating in patient care; (3) being able to communicate clearly; and (4) providing informed consent. No enrolled caregiver withdrew from the study. The requirement that patients had been successfully weaned from invasive mechanical ventilation for at least 72 consecutive hours was used to ensure that participants were recruited during an active transition stage, when family caregivers were expected to assume increasing caregiving responsibilities after transfer from the RICU.

Given the descriptive aim of this questionnaire‐based study, the sample size was determined pragmatically to ensure reasonably stable item‐level and domain‐level estimates rather than for hypothesis testing. The 22‐item scale was used as a practical planning reference and a minimum target of approximately 110 caregivers was considered sufficient for descriptive analysis. Allowing for potential incomplete responses, the target sample size was set at 128. During the study period, a total of 213 patient‐caregiver dyads were assessed for eligibility at the transfer‐preparation stage. Of these, 128 eligible caregivers were enrolled in the quantitative phase. Of the 85 cases not enrolled, 32 patients did not meet the clinical eligibility criteria, primarily because they had not been successfully weaned from invasive mechanical ventilation for ≥ 72 consecutive hours or were considered clinically unstable for transfer by the treating team; 28 cases had no eligible primary caregiver available for recruitment, because the primary caregiver was not consistently involved in care or was unavailable during the recruitment window; and 15 caregivers declined participation, citing time constraints, family burden or lack of interest. The remaining cases comprised transfer to another hospital before recruitment (*n* = 4), discharge against medical advice (*n* = 3) and communication barriers that precluded informed consent (*n* = 3).

#### 2.2.2 Qualitative Phase

Participants for the qualitative interviews were selected from the quantitative cohort using purposive sampling (Palinkas et al. 2015). To facilitate integration between the two phases, caregivers who had completed the quantitative survey and whose overall need scores fell within the highest‐scoring 30% of the quantitative sample were included in the eligible qualitative sampling pool (*n* = 38). This strategy was used to ensure that the qualitative phase could provide richer explanations for the highest‐priority needs identified in the quantitative analysis. Eleven caregivers were purposively selected and interviewed, with sampling continuing until data saturation was reached.

The sample size of 11 interviews was guided by the principle of data saturation, which was considered achieved when three consecutive interviews yielded no new themes (Guest et al. 2006). Although 11 is a relatively small sample by conventional qualitative standards, it is consistent with published mixed‐methods studies in critical care nursing that have used purposive selection from a larger quantitative cohort to explore extreme or high‐need cases (Choi et al. 2011). Importantly, the qualitative sample was not intended to represent the full spectrum of caregiver experiences but rather to provide in‐depth explanatory insights into the highest levels of perceived needs identified in the quantitative phase. The participants were deliberately selected from the highest‐scoring 30% of the quantitative sample based on overall need scores and this high‐need focus should be explicitly considered when interpreting the qualitative themes. Caregivers with lower levels of perceived need may have different experiences or priorities that are not captured in this analysis. Additionally, the interview duration (15–30 min) was relatively brief; however, the focused nature of the interviews, concentrating specifically on transfer‐related experiences, allowed for sufficient depth within this timeframe, particularly as participants had recently completed the quantitative survey and were familiar with the topic areas.

### 2.3 Data Collection

#### 2.3.1 Instruments

##### 2.3.1.1 Demographic and Clinical Information Questionnaire

A structured questionnaire was developed to collect demographic and clinical information. Patient data included age, sex, diagnosis, consciousness status, treatment modalities, length of RICU stay, time from tracheostomy to RICU transfer or discharge and transition destination. Caregiver data included age, sex, educational level, monthly household income, relationship to the patient, residential location, employment status, medical payment type, previous caregiving experience and previous formal training in tracheostomy care.

##### 2.3.1.2 Family Needs in ICU Transition Scale

Family caregivers' support needs were assessed using the Family Needs in ICU Transition Scale developed by Ye (2021). The scale was developed and validated in Chinese. It includes 22 items across five domains: caregiving skills (5 items), communication (5 items), instrumental support (5 items), emotional support (3 items) and information needs (4 items). Each item is rated on a 5‐point Likert scale ranging from 1 (not needed) to 5 (highly needed), with total scores ranging from 22 to 110. Higher scores indicate greater levels of perceived need. In the original development study, the scale showed acceptable psychometric properties, including a scale‐level content validity index of 0.898, a Cronbach's α of 0.895 for the total scale and a split‐half reliability coefficient of 0.807 (Ye 2021). In the current study, the Cronbach's α coefficient for the total scale was 0.903 and the coefficients for the five domains ranged from 0.853 to 0.886, indicating good internal consistency in the present sample. This scale was considered particularly suitable for the present study because, compared with more general family‐needs instruments, it specifically focuses on support needs during the ICU transition period.

##### 2.3.1.3 Semi‐Structured Interview Guide

Based on the quantitative findings, a semi‐structured interview guide was developed and refined through expert consultation (Turner III 2010). The guide focused on caregivers' experiences during patient transfer, perceived challenges in caregiving, communication with healthcare providers and emotional needs.

#### 2.3.2 Procedures

Quantitative data were collected through face‐to‐face administration of the structured questionnaire by trained nurse researchers. Caregivers self‐completed the questionnaires under the guidance of the researchers to ensure consistency. Researchers provided clarification when needed but did not influence participants' responses. The questionnaires were administered to primary caregivers within 24–48 h before the patient's transfer from the RICU to the general ward or home. All participating caregivers had already been involved in the patient's daily care before the survey.

For the qualitative phase, individual interviews were conducted after participants had completed the quantitative survey and following the patients' transfer to the feneral ward or home. Interviews were conducted either in a private room at the hospital or via secure video call, depending on participant preference. The interviews lasted between 15 and 30 min. They were audio‐recorded with participants' consent, transcribed verbatim for analysis and anonymized using unique identifiers (e.g., F1–F11).

The research team comprised two registered nurses with backgrounds in critical care and one researcher with expertise in qualitative methodology. All team members held master's degrees in nursing and had prior experience conducting health‐related qualitative research. Given that the primary data collectors (Y.W. and Y.S.) are RICU nurses who interact regularly with family caregivers in their clinical roles, we recognised that our professional backgrounds could shape both data collection and interpretation. To mitigate potential bias, the following strategies were employed: (1) interviewers explicitly introduced themselves as researchers rather than clinical staff at the start of each interview and emphasised that participants' responses would not affect their or their family members' care; (2) reflexive memos were maintained after each interview to document the interviewer's impressions, assumptions, and potential emotional responses; (3) independent coding by two researchers (Y.W. and Z.W.) was followed by consensus discussion, with discrepancies resolved through reference to the raw data rather than clinical intuition; (4) the interview guide was reviewed by Z.W., who was not involved in direct patient care, to reduce clinical framing of questions; and (5) member checking was conducted with all 11 participants to ensure that the interpreted themes aligned with their intended meanings. Despite these precautions, we acknowledge that complete elimination of researcher bias is not possible, and the interpretive lens of clinically trained researchers may have influenced the focus on skill‐ and communication‐related themes. We recommend that readers consider this context when interpreting the findings.

### 2.4 Data Analysis

Quantitative data were analysed using SPSS version 25.0 (IBM Corp., Armonk, NY, USA). For the quantitative survey data, the proportion of missing item‐level responses was less than 5% for each survey item. Given the low proportion of missing data, missing values for continuous variables were handled using mean imputation, and missing values for categorical variables were handled using mode imputation. No missing data were present for the demographic and clinical variables included in the analysis. Listwise deletion was not applied. Descriptive statistics, including means, standard deviations, frequencies, and percentages, were used to summarize demographic characteristics and caregiver needs. The overall level of need was expressed as the mean item score across the 22 items of the Family Needs in ICU Transition Scale on the original 1–5 Likert scale. For subgroup analyses, independent sample *t*‐tests were used to compare overall need scores between two groups (rural vs. urban residence, with vs. without prior caregiving experience, college or above vs. below college education, and transfer to general ward vs. direct home discharge). One‐way analysis of variance (ANOVA) was used for comparisons across three or more groups (relationship to patient: spouse, child or other). Effect sizes were reported as Cohen's d for *t*‐tests and *η* ^2^ for ANOVA. All subgroup analyses were exploratory and were not adjusted for multiple comparisons.

Qualitative data were analysed using Colaizzi's seven‐step phenomenological method (Colaizzi 1978). NVivo version 12 (QSR International, Melbourne, Australia) was used to assist with data organization and coding. Two researchers with prior experience in qualitative health research independently coded the interview transcripts, and discrepancies were resolved through discussion until consensus was reached. Coding records and analytic memos were maintained during data analysis to support the audit trail and development of the thematic framework. After the thematic framework had been developed, member checking was conducted with all 11 interview participants by returning a summary of the themes for confirmation and feedback. The researchers were clinicians working in critical care‐related settings. To reduce potential interpretive bias, coding and theme development were discussed iteratively and grounded in the interview data rather than prior clinical assumptions.

After the quantitative and qualitative analyses were completed, the two sets of findings were integrated at the interpretation stage. Qualitative themes were compared with the quantitative domain rankings and high‐priority items to identify convergence and to provide contextual explanations for the most prominent caregiver needs during the transition process.

## 3 Results

### 3.1 Quantitative Findings

#### 3.1.1 Demographic Characteristics of Tracheostomized Patients and Caregivers

A total of 128 tracheostomized patients were included in this study. The main clinical diagnoses were severe pneumonia (42.2%), acute exacerbation of chronic obstructive pulmonary disease (AECOPD, 28.9%), respiratory failure (19.5%), and septic shock (9.4%). The mean length of stay in the RICU was 21.6 ± 8.3 days, and the mean time from tracheostomy to the transition was 15.3 ± 6.7 days. At the time of transition, 67.2% of patients were alert, 23.4% had mild impairment of consciousness, and 9.4% were drowsy. All patients had been successfully weaned from mechanical ventilation for at least 72 h before the transition and were hemodynamically stable.

All 128 questionnaires were retained for analysis after imputation of the small proportion of missing item‐level responses. Most caregivers were spouses (52.3%) or children (38.2%) of the patients. Their ages ranged from 22 to 71 years, with a mean age of 46.5 ± 12.3 years. Most were female (65.6%). Regarding educational background, 61.7% had attained at least a college degree. Approximately 63.3% resided in rural areas, and 78.9% reported a monthly household income below RMB 20,000. Most patients (93.0%) were covered by health insurance. In addition, 40.6% (52/128) of caregivers were employed, whereas 59.4% (76/128) were unemployed or retired. A total of 35.2% (45/128) had prior caregiving experience for older or chronically ill family members, while 64.8% (83/128) had no prior caregiving experience. Only 14.1% (18/128) had received formal training in tracheostomy care, whereas 85.9% (110/128) had not received formal tracheostomy care training.

#### 3.1.2 Assessment of Caregiver Needs

The overall mean item score for caregiver needs was 4.10 ± 0.85 on the 5‐point Likert scale, indicating a generally high level of perceived need. Among the five domains, caregiving skills scored the highest (4.25 ± 0.80), followed by information needs (4.18 ± 0.84) and communication with healthcare providers (4.15 ± 0.81). Instrumental support (3.99 ± 0.83) and emotional support (3.84 ± 0.96) were rated comparatively lower. As shown in Table 1 (where High Need *n* (%) represents the number and percentage of caregivers scoring 4 or 5 for each item), the highest‐rated individual item was the need to be informed when patients require urgent medical attention (4.73 ± 0.50), followed by knowing key bedside observations (4.48 ± 0.77) and understanding the patient's suitability for transfer (4.34 ± 0.75).

**TABLE 1:** Full ranking of all 22 items in the Family Needs in ICU Transition Scale (*n* = 128).

Rank	Item	Domain	High need *n* (%)	Mean ± SD
1	Being informed about conditions requiring immediate notification of medical staff	Caregiving Skills	123 (96.0%)	4.73 ± 0.50
2	Knowing key observations during bedside care	Caregiving Skills	111 (86.7%)	4.48 ± 0.77
3	Understanding whether the patient is suitable for transfer	Information Needs	112 (87.5%)	4.34 ± 0.75
4	Receiving honest answers from medical staff	Communication Needs	107 (83.6%)	4.31 ± 0.93
5	Obtaining clear and understandable explanations	Communication Needs	100 (78.1%)	4.16 ± 0.86
6	Understanding the next stage of treatment	Information Needs	106 (82.8%)	4.13 ± 1.06
7	Daily communication with healthcare providers regarding the patient's condition	Communication Needs	99 (77.3%)	4.09 ± 0.80
8	Being informed promptly of changes in the treatment plan	Communication Needs	95 (74.2%)	4.07 ± 1.02
9	Learning to provide basic comfort care (e.g., hygiene care and massage)	Caregiving Skills	96 (75.0%)	4.06 ± 0.85
10	Being instructed on how to assist patients with early functional rehabilitation exercises	Caregiving Skills	92 (71.9%)	3.99 ± 0.88
11	Expecting ICU nurses to organize a family care meeting before patient transfer	Instrumental Support	90 (70.3%)	3.99 ± 0.86
12	Expecting provision of a family caregiving handbook for the ICU transition period	Instrumental Support	88 (68.8%)	3.98 ± 0.84
13	Being informed about dietary precautions for the patient	Caregiving Skills	85 (66.4%)	3.95 ± 0.87
14	Being able to visit and become familiar with the general ward environment before transfer from ICU	Information Needs	83 (64.8%)	3.92 ± 0.89
15	Expecting the department to establish peer‐support groups for families with similar diseases	Communication Needs	81 (63.3%)	3.89 ± 0.90
16	Being informed about the physicians and nurses responsible for the patient in the general ward	Information Needs	79 (61.7%)	3.87 ± 0.91
17	Expecting healthcare providers to recognize the role of family members in patient recovery	Emotional Support	77 (60.2%)	3.85 ± 0.94
18	Expecting healthcare providers to help alleviate doubts and fears about the future	Emotional Support	75 (58.6%)	3.82 ± 0.97
19	Expecting healthcare providers to encourage patients and families by sharing successful cases	Emotional Support	73 (57.0%)	3.80 ± 0.98
20	Expecting that, under special circumstances, two family members may be allowed to provide care simultaneously	Instrumental Support	71 (55.5%)	3.78 ± 0.99
21	Expecting healthcare providers to also be concerned about family members' health	Instrumental Support	69 (53.9%)	3.76 ± 1.00
22	Expecting the hospital cafeteria to provide more palatable meals	Instrumental Support	67 (52.3%)	3.74 ± 1.01

*Note:* High Need *n* (%) refers to the number and percentage of caregivers who rated the item as 4 (Fairly needed) or 5 (Highly needed) on the 5‐point Likert scale.

#### 3.1.3 Exploratory Subgroup Analyses

Subgroup analyses were conducted to examine differences in caregiver need levels across key demographic and clinical characteristics (Table 2). Caregivers residing in rural areas reported significantly higher overall needs than their urban counterparts (4.28 ± 0.78 vs. 3.82 ± 0.91, *t* = 2.89, *p* = 0.004, Cohen's d = 0.54). Similarly, caregivers with no prior caregiving experience reported higher needs than those with prior experience (4.31 ± 0.82 vs. 3.72 ± 0.85, *t* = 3.74, *p* < 0.001, d = 0.70). No significant differences were observed by educational level (college degree or above vs. below college: 4.02 ± 0.88 vs. 4.22 ± 0.80, *t* = 1.29, *p* = 0.199). Regarding the relationship to the patient, spouses reported higher needs than children (4.28 ± 0.76 vs. 3.92 ± 0.95, *F* = 3.17, *p* = 0.045, *η* ^2^ = 0.04), although this difference did not reach statistical significance. Notably, caregivers of patients discharged directly home reported higher overall needs than those of patients transferred to the general ward (4.41 ± 0.72 vs. 3.98 ± 0.88, *t* = 2.68, *p* = 0.008, d = 0.53), suggesting that transition destination may be associated with caregiver support needs.

**TABLE 2:** Subgroup comparisons of caregiver needs by demographic and clinical characteristics.

Characteristic	*n* (%)	Mean ± SD	*t*/*F*	*p*	Effect size
Residence					
Urban	47 (36.7)	3.82 ± 0.91	2.89	0.004	d = 0.54
Rural	81 (63.3)	4.28 ± 0.78			
Caregiving experience					
Yes	45 (35.2)	3.72 ± 0.85	3.74	< 0.001	d = 0.70
No	83 (64.8)	4.31 ± 0.82			
Education					
College or above	79 (61.7)	4.02 ± 0.88	1.29	0.199	—
Below college	49 (38.3)	4.22 ± 0.80			
Relationship					
Spouse	67 (52.3)	4.28 ± 0.76	2.48	0.088	*η* ^2^ = 0.04
Child	49 (38.3)	3.92 ± 0.95			
Other	12 (9.4)	3.85 ± 0.82			
Transition destination					
General ward	89 (69.5)	3.98 ± 0.88	2.68	0.008	d = 0.53
Home	39 (30.5)	4.41 ± 0.72			

### 3.2 Qualitative Findings

#### 3.2.1 Demographic and Clinical Characteristics of Interview Participants

Eleven family caregivers from the high‐need qualitative sampling pool were purposively selected for qualitative interviews. Participants included eight females and three males, aged 22–71 years (mean: 51.3 ± 17.8 years). Most were either spouses (54.5%) or children (36.4%) of the patients. Educational attainment varied, with 54.5% having completed college or above. Seven participants (63.6%) resided in rural areas. The most common patient diagnosis was pulmonary infection (6/11), followed by respiratory failure (2/11). Table 3 presents detailed demographic and clinical information.

**TABLE 3:** Demographic and clinical characteristics of qualitative interview participants (*n* = 11).

ID	Gender	Age	Relationship	Education	Residence	Income	Payment	Patient Age	Patient gender	Diagnosis	Consciousness	Treatment	Interview duration	Mode
F1	Female	68	Spouse	High school	Rural	< 10,000	Insurance	76	Male	Septic shock	Drowsy	ECMO + CRRT + Anti‐infection + Nutrition	20 min	Face‐to‐face
F2	Female	47	Daughter	College	Urban	10,000‐20,000	Insurance	79	Female	Pulmonary infection	Clear	Anti‐infection + Nutrition + Gastric protection	16 min	Face‐to‐face
F3	Female	31	Daughter	Master	Urban	> 20,000	Insurance	57	Male	AECOPD	Clear	Anti‐infection + Nutrition	15 min	Video call
F4	Female	68	Spouse	Junior high	Rural	< 10,000	Insurance	73	Male	Pulmonary infection	Light coma	Anti‐infection + Nutrition	17 min	Face‐to‐face
F5	Female	35	Spouse	Bachelor	Urban	10,000‐20,000	Insurance	37	Male	Pulmonary infection	Clear	Anti‐infection + Nutrition	15 min	Face‐to‐face
F6	Female	68	Spouse	High school	Rural	< 10,000	Insurance	76	Male	Severe pneumonia	Light coma	Anti‐infection + Nutrition + Intermittent CRRT	15 min	Face‐to‐face
F7	Male	22	Grandson	Bachelor	Rural	< 10,000	Insurance	69	Male	Respiratory failure	Clear	Anti‐infection + Nutrition	18 min	Face‐to‐face
F8	Female	41	Daughter	College	Rural	10,000‐20,000	Insurance	64	Female	Respiratory failure	Deep coma	Anti‐infection + Nutrition	16 min	Video call
F9	Female	67	Spouse	Bachelor	Urban	> 20,000	Self‐pay	74	Male	Pulmonary infection	Clear	Anti‐infection + Nutrition + CRRT	15 min	Face‐to‐face
F10	Male	46	Son	College	Rural	> 20,000	Insurance	76	Male	Pulmonary infection	Deep coma	Anti‐infection + Nutrition	15 min	Face‐to‐face
F11	Male	71	Spouse	Primary school	Rural	< 10,000	Insurance	77	Female	Pulmonary infection	Clear	Anti‐infection + Nutrition	16 min	Face‐to‐face

Abbreviations: AECOPD, acute exacerbation of chronic obstructive pulmonary disease; CRRT, continuous renal replacement therapy; ECMO, extracorporeal membrane oxygenation.

#### 3.2.2 Themes Identified From Interviews

A total of five major themes emerged from the qualitative interviews, reflecting the multifaceted challenges and unmet needs experienced by caregivers in this high‐need subgroup (those whose overall need scores fell within the highest‐scoring 30% of the quantitative sample). These themes should be interpreted specifically within this high‐need context and do not necessarily represent the experiences of caregivers with lower or moderate levels of perceived need. The themes are presented below with illustrative quotations.

##### 3.2.2.1 Theme 1: Need for Timely and Detailed Information Before and During the Transfer Process

Caregivers in this high‐need subgroup emphasized the need for timely and detailed information before and during the patient's transfer, including clarification of the transfer process, preparation requirements, and immediate care arrangements. Many described the transfer notification as abrupt and disorienting. One daughter, whose mother was being transferred from the RICU, recalled: ‘When I got the transfer notice, I felt completely overwhelmed and had no idea what to prepare first. Everything was just chaotic.’ (F2) This sense of being ill‐prepared was echoed by another participant, who expressed anxiety about the practical aspects of the transfer itself: ‘I was worried about whether the oxygen supply during transport would be sufficient. No one clearly explained what to expect.’ (F4) A third caregiver described the logistical challenges of the sudden transition: ‘The transfer notification came too suddenly. I had no time to prepare properly and ended up scrambling to pack everything.’ (F5) These accounts collectively suggest that caregivers received insufficient advance warning and preparation for the transfer process, contributing to stress and confusion at an already vulnerable time.

##### 3.2.2.2 Theme 2: Urgent Need for Professional Guidance on Post‐Transfer Care Skills

Caregivers in this high‐need subgroup consistently articulated a strong need for practical, hands‐on guidance in caregiving skills after patient transfer. The technical nature of tracheostomy care emerged as a particular source of anxiety. One spouse of a patient with a newly placed tracheostomy tube expressed her apprehension candidly: ‘Now that he has the tracheostomy tube, I really don't know how to handle it properly.’ (F1) Physical care tasks were also a concern, particularly for caregivers who lacked the physical strength or training to manage repositioning and feeding. A daughter caring for her elderly mother commented: ‘I can't manage turning her over on my own. I just don't have the strength.’ (F2) Similarly, a younger caregiver voiced uncertainty about basic nutritional care: ‘I wonder whether the food he's getting is enough, and whether I can give him water. I really don't know.’ (F3) Beyond these general care concerns, participants also highlighted the complexity of managing enteral feeding tubes. One participant noted: ‘I'm still confused about how to manage the nasoenteric feeding tube.’ (F10) Another added: ‘Recording intake and output, flushing the feeding tube… these tasks are complicated for me, and I worry about causing blockages.’ (F11) These quotations illustrate a pervasive lack of confidence in performing both basic and technically complex care tasks, a gap that educational interventions could potentially address.

##### 3.2.2.3 Theme 3: Expectation for Ongoing Communication and Information Transparency After Transfer

After transfer, caregivers in this high‐need subgroup reported persistent challenges in obtaining timely information and maintaining communication with healthcare providers. Concerns centered on medication administration, medical device management, and examination scheduling. One caregiver described the confusion that arose when treatment changes were not explained: ‘When new medications were administered, no one explained what they were for. I felt lost and helpless.’ (F2) Another participant voiced anxiety about the respiratory equipment: ‘I worried about how the respiratory equipment was working and whether it made him uncomfortable, but no one explained it.’ (F4) The difficulty of accessing busy clinical staff was a recurring theme; one caregiver observed: ‘Doctors are always busy, and it's hard to get updates unless we actively chase them. When I asked about tracheostomy care, they didn't have time to answer all my questions.’ (F5) Even routine procedures such as patient transport for examinations were perceived as poorly communicated: ‘Sometimes patients were sent out for examinations without informing us in advance. I wished they could have told us earlier so that I could prepare my son to accompany her.’ (F11) These accounts point to systematic gaps in information transparency and caregiver inclusion in care planning, even after the transition had occurred.

##### 3.2.2.4 Theme 4: Request for Caregiver Educational Materials

In addition to direct instruction, caregivers in this high‐need subgroup expressed a strong preference for supplementary educational materials, such as written manuals, posters or simple instructional videos. Several participants noted that verbal explanations alone were insufficient, particularly given the stress of the caregiving situation. A grandson caring for his elderly grandfather explained: ‘If we had a handbook showing step‐by‐step what to watch for and how to act, it would help us a lot.’ (F7) Another participant, a daughter of a patient, emphasized the fallibility of memory under stress: ‘Sometimes after listening to verbal instructions, I forget the details. Having written materials would make me feel more secure.’ (F2) These comments suggest that educational materials should be designed not as a replacement for hands‐on teaching but as a complement that caregivers can consult when anxiety or fatigue impairs recall of verbal instructions.

##### 3.2.2.5 Theme 5: Desire for Emotional Support and Encouragement

Emotional support emerged as a cross‐cutting concern, with caregivers in this high‐need subgroup describing the psychological toll of their responsibilities and the importance of empathic communication from healthcare providers. One participant described the physical and emotional exhaustion of continuous caregiving: ‘Taking care of her is truly exhausting. A few words of encouragement make a big difference.’ (F8) Another articulated the sense of isolation that can accompany the caregiving role: ‘Sometimes I feel like I'm fighting alone. Positive feedback from doctors or nurses would really help.’ (F9) These statements highlight that, beyond informational and practical support, caregivers derive meaningful reassurance from simple interpersonal gestures of encouragement and validation.

The qualitative and quantitative findings showed clear convergence. The themes of practical caregiving guidance, timely transfer‐related information, and effective provider communication corresponded closely to the highest‐scoring quantitative domains of caregiving skills, information needs, and communication with healthcare providers. In addition, the qualitative findings extended the quantitative results by illustrating how caregivers experienced these needs in practice, particularly with respect to educational materials and emotional support during the transition period.

### 3.3 Integration of Quantitative and Qualitative Findings

To facilitate the integration of the two data strands, we constructed a joint display table (Table 4) to directly compare the quantitative domain scores and highest‐ranked items with the corresponding qualitative themes and representative quotes. This side‐by‐side presentation illustrates the convergence and complementary insights between the quantitative and qualitative findings.

**TABLE 4:** Joint display of quantitative and qualitative findings.

Quantitative domain (mean ± SD)	Top quantitative item(s) within domain	Corresponding qualitative theme	Illustrative quote
Caregiving skills (4.25 ± 0.80)	‘Being informed about conditions requiring immediate notification’ (4.73 ± 0.50); ‘Knowing key bedside observations’ (4.48 ± 0.77)	Theme 2: Urgent need for professional guidance on post‐transfer care skills	‘Now that he has the tracheostomy tube, I really don't know how to handle it properly.’ (F1)
Information needs (4.18 ± 0.84)	‘Understanding whether the patient is suitable for transfer’ (4.34 ± 0.75); ‘Understanding the next stage of treatment’ (4.13 ± 1.06)	Theme 1: Need for timely and detailed information before and during transfer	‘When I got the transfer notice, I felt completely overwhelmed and had no idea what to prepare first.’ (F2)
Communication with HCPs (4.15 ± 0.81)	‘Receiving honest answers’ (4.31 ± 0.93); ‘Clear explanations’ (4.16 ± 0.86)	Theme 3: Expectation for ongoing communication and information transparency	‘Doctors are always busy, and it's hard to get updates unless we actively chase them.’ (F5)
Instrumental support (3.99 ± 0.83)	‘Expecting ICU nurses to organize a family care meeting’ (3.99 ± 0.86); ‘Expecting a family caregiving handbook’ (3.98 ± 0.84)	Theme 4: Request for caregiver educational materials	‘If we had a handbook showing step‐by‐step what to watch for and how to act, it would help us a lot.’ (F7)
Emotional support (3.84 ± 0.96)	‘Recognizing the role of family members’ (3.85 ± 0.94); ‘Alleviating doubts and fears’ (3.82 ± 0.97)	Theme 5: Desire for emotional support and encouragement	‘Sometimes I feel like I'm fighting alone. Positive feedback from doctors or nurses would really help.’ (F9)

## 4 Discussion

This study provides quantitative evidence on support needs in the full sample and in‐depth qualitative insight into the experiences of a high‐need subgroup during the transition of tracheostomized patients from the RICU. Quantitative results indicated that caregiving competence, information acquisition, and communication with healthcare professionals were the top priorities. Qualitative analysis of this high‐need subgroup further revealed five major themes: the need for detailed information regarding patient transfer, practical caregiving guidance, improved communication, accessible educational resources, and emotional support. These findings underscore the multifaceted unmet needs faced by caregivers during the post‐RICU transition and highlight the need for more targeted, family‐centred support during this period (Choi et al. 2011).

The high demand for caregiving skill training observed in this study is consistent with previous research showing that family caregivers of critically ill patients often feel underprepared for complex post‐ICU care responsibilities and may experience substantial burden during recovery and transition periods (Fumis et al. 2015). In the context of tracheostomized patients, these findings highlight the importance of structured caregiver education to support safe and confident participation in care after RICU discharge (Abe et al. 2013). The findings should be understood within China's cultural and healthcare context, where family caregivers may assume substantial hands‐on caregiving responsibilities during and after hospitalization. The predominance of rural‐residing caregivers and the relatively limited household income reported by many participants may also have shaped access to health information and post‐discharge support. Because this study did not directly compare countries or healthcare systems, cross‐national differences should be interpreted cautiously. These contextual considerations indicate that transitional care interventions developed in other healthcare systems may require local adaptation before implementation in China.

It is important to emphasize that the qualitative findings reported in this study were derived exclusively from caregivers with the highest levels of perceived needs (the highest‐scoring 30% of the quantitative sample based on overall need scores). While these themes provide rich, in‐depth explanations for the most urgent unmet needs, they are not intended to represent the full spectrum of experiences among all family caregivers of patients with tracheostomies. Caregivers with lower need scores may have different priorities, concerns or coping strategies that are not captured in our qualitative analysis. Therefore, the qualitative themes should be interpreted as reflecting the perspectives of this specific high‐need subgroup rather than the general caregiver population.

Communication gaps were another major concern identified in both quantitative and qualitative results. Caregivers frequently reported insufficient updates and limited participation in care‐related decisions, which contributed to feelings of helplessness. Previous studies have shown that family‐support and family‐centred communication strategies in intensive care settings can improve families' experiences of care and strengthen communication processes (White et al. 2018; Davidson et al. 2017). Consistent with these findings, our results suggest the value of more structured communication during the transition process. This may include clearer transfer‐related explanations, timely updates on treatment plans, and designated opportunities for caregivers to raise questions. Such approaches may help address the difficulty caregivers described in obtaining timely information from busy clinical staff.

Among the high‐need interview subgroup, the need for practical educational materials was also widely expressed. Written guides, visual aids, and mobile‐accessible content were viewed as useful complements to verbal instruction. Our findings are also consistent with previous research indicating that family caregivers value clear written information and practical educational support during complex care transitions and home‐based care after discharge (Al‐Mutair et al. 2013; Wendler and Rid 2011). Such materials should be introduced prior to discharge and adapted to the caregivers' literacy level and learning preferences.

Within this high‐need subgroup, emotional burden emerged as a cross‐cutting theme in the qualitative results, with caregivers describing stress, fatigue, and feelings of isolation during the post‐RICU transition. Consistent with previous research showing that family members of critically ill patients commonly experience substantial emotional distress during and after ICU care (Smith et al. 2025; Kentish‐Barnes et al. 2017), these results suggest that emotional support should be incorporated into transitional care. Empathetic communication, reassurance, and timely identification of caregivers who may require additional support may be particularly important during this stage. Given that many caregivers in this study were from rural areas or reported relatively limited household income, caregiver support strategies should also consider potential differences in access to health information and post‐discharge resources.

Additionally, concerns about the transfer process itself were prominent among this high‐need subgroup. Caregivers often perceived the transition as abrupt and poorly coordinated. This observation is in line with earlier post‐ICU research suggesting that the period following ICU discharge can be stressful and challenging for patients and families, underscoring the importance of careful transition planning and continuity of support (Tilburgs et al. 2025). Early involvement of caregivers in discharge planning, including discussions of patient stability, transfer logistics, and post‐transfer expectations, is critical for improving preparedness.

The demographic profile of the sample also warrants consideration. A substantial proportion of caregivers were from rural areas and reported relatively limited household income, which may influence access to educational materials, confidence in navigating healthcare information, and the availability of post‐discharge support resources. These contextual factors should be considered when developing caregiver support strategies.

These findings suggest several concrete nursing actions during the RICU transition. First, a structured transfer briefing checklist may help ensure that caregivers receive consistent information on transfer timing, immediate precautions, and post‐transfer expectations. Second, caregiver education may be organised as a stepwise pathway covering tracheostomy‐related observation, emergency warning signs, feeding, positioning, and basic comfort care, with return demonstration where feasible. Third, written manuals, bedside posters or short mobile‐accessible videos tailored to caregivers' literacy levels may reinforce verbal teaching. Fourth, scheduled opportunities for family updates, such as designated daily communication times, may improve information transparency and reduce uncertainty. Finally, caregivers with marked distress may benefit from early emotional assessment, supportive counselling or referral when needed.

The joint display (Table 4) demonstrates how the qualitative themes from the high‐need subgroup converged with and extended the quantitative domain scores. The three highest‐scoring domains, caregiving skills, information needs, and communication, were each echoed by corresponding qualitative themes. Importantly, the qualitative findings illuminate the mechanisms underlying these scores: caregivers' urgent demand for skill training reflected specific anxieties about performing tracheostomy‐related procedures without adequate hands‐on guidance, while the high priority assigned to communication needs stemmed from feeling excluded from care decisions and struggling to obtain timely updates from busy staff, suggesting that ‘communication need’ may represent a deeper need for inclusion and information transparency. The qualitative data also extended the quantitative findings by revealing contextual and emotional dimensions that the survey alone could not capture. Although emotional support and instrumental support scored relatively lower, interviews revealed they remained critically important, particularly for caregivers with limited social support or those transitioning directly home, suggesting that quantitative scores may underestimate the salience of emotional needs when measured alongside more tangible needs. Furthermore, the recurring demand for practical educational materials, rated lower in the survey, emerged from caregivers' descriptions of how stress and fatigue impaired recall of verbal instructions, indicating that interventions should incorporate written materials and visual aids as essential complements to verbal teaching. Together, these integrated findings suggest that effective transitional care interventions must address not only skill‐building and information provision, but also communication processes that shape caregivers' sense of inclusion and the emotional support that sustains their confidence during this highly stressful period.

The findings of this study should be interpreted within the context of the Chinese healthcare system, where family caregivers are traditionally expected to assume substantial caregiving responsibilities during and after hospitalization. In other healthcare systems, particularly those with more formalized post‐discharge support structures or different patterns of family involvement, the specific needs identified here may manifest differently or with varying intensity. For instance, in settings where skilled nursing facilities or home health services are more readily available, caregivers' needs for practical skill training and instrumental support might be less pronounced, whereas in settings with more limited inpatient visitation policies, needs related to communication and information access could be even greater. Similarly, the high proportion of rural‐residing caregivers in our sample (63.3%) may reflect a population with reduced access to health information and post‐discharge resources, meaning that the needs identified here may be particularly salient for underserved populations but may not fully represent urban caregivers with better health literacy and service access. These contextual factors should be considered when applying our findings to other settings, and future multicenter studies are needed to examine how regional, cultural, and health system variations shape family caregivers' transition needs.

### 4.1 Strengths and Limitations

This mixed‐methods study provides integrated insights into the support needs of family caregivers during the post‐RICU transition of tracheostomized patients. A key strength of the study is its sequential explanatory design, which combines quantitative assessment with qualitative exploration to provide a more comprehensive understanding of caregivers' support needs.

Several limitations of this study warrant careful consideration. First, the single‐center design and consecutive sampling strategy limit the generalizability of the findings. The study was conducted in a tertiary respiratory ICU in China, where care delivery models, family involvement patterns, and healthcare resources may differ substantially from those in other settings. The findings may not be transferable to non‐tertiary hospitals, other geographic regions or countries with different healthcare systems and cultural norms around family caregiving. Second, the quantitative analysis was primarily descriptive and did not include multivariable adjustment. Although subgroup analyses were performed, these were exploratory and were not adjusted for multiple comparisons; therefore, the observed associations should be interpreted cautiously. The reported effect sizes provide some indication of the magnitude of these differences, but confirmatory studies with larger samples and prespecified hypotheses are needed to validate these findings. Third, the qualitative interviews were conducted only with caregivers reporting the highest‐scoring 30% of the quantitative sample based on overall need scores. While this sampling strategy was intentional to maximize explanatory depth regarding the most urgent needs, it means that the qualitative findings do not reflect the experiences of caregivers with lower or moderate levels of perceived need. The qualitative themes identified here may, therefore, represent the ‘worst‐case’ scenario rather than the full spectrum of caregiver experiences. Fourth, social desirability bias may have affected responses in both the survey and interviews, as data collection was conducted by researchers who were also RICU nurses. Although we explicitly emphasized the research nature of the data collection and assured participants that their responses would not affect patient care, some caregivers may have been reluctant to report negative experiences or criticize healthcare providers. Fifth, the study did not distinguish between transition to the general ward and transition to home in the primary analysis, despite evidence from subgroup analyses that transition destination may influence caregiver needs. The relatively small number of caregivers in the home‐transition group (*n* = 39) limited our ability to conduct stratified analyses with adequate statistical power. Sixth, patient clinical outcomes were not measured, limiting our ability to link caregiver needs to patient outcomes. It remains unclear whether the high levels of caregiver need identified here translate into adverse patient outcomes or whether addressing these needs would improve patient recovery. Seventh, the cross‐sectional design captures caregiver needs at a single time point (24–48 h before transfer) and does not capture how needs may evolve over the post‐discharge trajectory. Future longitudinal studies are needed to examine changes in caregiver needs and the timing of interventions. Finally, the use of a single validated scale, while appropriate for this study, may not capture all dimensions of caregiver need. Domains such as financial burden, spiritual support, and impact on the caregiver's own health were not fully addressed by the scale and may represent important unmet needs not identified in this study.

Future research should use multicenter designs, include inferential analyses, distinguish between different transition destinations, and examine longer‐term caregiver needs and patient outcomes.

## 5 Conclusion

Family caregivers of tracheostomized patients face substantial support needs during RICU transitions, particularly in caregiving skills, communication, information access, and emotional support. Tailored, family‐centered interventions may enhance caregiver readiness and strengthen continuity of transitional care. Further research should develop and evaluate scalable discharge models that systematically integrate caregiver education, communication, and psychosocial support into routine transitional care.

## Author Contributions

**Ying Wei:** software, data curation, supervision. **Zhen Wang:** visualization, project administration, software, supervision. **Ying Sun:** project administration, visualization, conceptualization, methodology. **Muli Zhu:** writing – original draft, writing – review and editing.

## Funding

The authors have nothing to report.

## Ethics Statement

The study was approved by the Institutional Review Board of General Hospital of the Eastern Theatre Region (now knows as Jinling Hospital, Affiliated Hospital of Medical School, Nanjing University) (Approval No. 2024DZKY‐062‐01). Written informed consent was obtained from all participants prior to data collection. Participants were informed that their participation was entirely voluntary, and they were assured of the confidentiality and anonymity of their responses. Audio files and interview transcripts were stored in password‐protected files accessible only to the core research team, and all transcripts were anonymised using unique identifiers (e.g., F1–F11) before analysis. All procedures were conducted in accordance with the ethical standards of the institutional and/or national research committee and with the 1964 Helsinki Declaration and its later amendments or comparable ethical standards.

## Conflicts of Interest

The authors declare no conflicts of interest.

## Data Availability

The data that support the findings of this study are available from the corresponding author upon reasonable request, with access restricted by ethical considerations to protect participant privacy.
